# A Rare Case of Polysplenia Syndrome Associated with Severe Cardiac Malformations and Congenital Alveolar Dysplasia in a One-Month-Old Infant: A Complete Macroscopic and Histopathologic Study

**DOI:** 10.3390/jcdd9050135

**Published:** 2022-04-27

**Authors:** Cosmin Ioan Mohor, Sorin Radu Fleaca, Alexandra Oprinca Muja, George Calin Oprinca, Mihai Dan Roman, Radu Chicea, Adrian Gheorghe Boicean, Horatiu Dura, Ciprian Tanasescu, Nicolas Catalin Ionut Ion, Mihai Faur, Ciprian Ionut Bacila, Florina Batar, Calin Ilie Mohor

**Affiliations:** 1 Department of Basic Science, Faculty of Medicine, Lucian Blaga University of Sibiu, 550024 Sibiu, Romania; cosmin.mohor@ulbsibiu.ro (C.I.M.); lilioaraalexandra.muja@ulbsibiu.ro (A.O.M.); georgecalin.oprinca@ulbsibiu.ro (G.C.O.); horatiu.dura@ulbsibiu.ro (H.D.); calin.mohor@ulbsibiu.ro (C.I.M.); 2 Department of Surgery, Faculty of Medicine, Lucian Blaga University of Sibiu, 550024 Sibiu, Romania; mihai.roman@ulbsibiu.ro (M.D.R.); radu.chicea@ulbsibiu.ro (R.C.); ciprian.tanasescu@ulbsibiu.ro (C.T.); nicolascatalinionut.ion@ulbsibiu.ro (N.C.I.I.); mihai.faur@ulbsibiu.ro (M.F.); 3 Department of Medicine, Faculty of Medicine, Lucian Blaga University of Sibiu, 550024 Sibiu, Romania; adrian.boicean@ulbsibiu.ro (A.G.B.); ciprian.bacila@ulbsibiu.ro (C.I.B.); florina.batar@ulbsibiu.ro (F.B.)

**Keywords:** polysplenia syndrome, left atrial isomerism, congenital alveolar dysplasia, hepatic fibrosis, infantile lactic acidosis, complete atrioventricular canal defect, truncus arteriosus, vena cava duplication, gall bladder agenesis, intestinal inversion

## Abstract

Polysplenia syndrome represents a type of left atrial isomerism characterized by multiple small spleens, often associated with cardiac malformations and with *situs ambiguus* of the abdominal organs. The case presented is of a one-month-old male infant, weighing approximately 3000 g, born at the County Clinical Emergency Hospital of Sibiu, who was hospitalized from birth until death. The patient suffered cardio-respiratory arrest due to severe hypoxia and septicemia on the background of a series of complex cardiac malformations associated with congenital abdominal organ anomalies. Examination of the body revealed a common atrium with complete atrioventricular canal defect, left ventricular hypertrophy, right ventricle hypoplasia, truncus arteriosus, superior vena cava duplication, bilobation of the lungs, situs ambiguous of the abdominal organs with right-sided stomach, a midline liver, gall bladder agenesis, multiple right-sided spleens and complete inversion of the intestines and pancreas. Histopathology concluded that the patient suffered cardiac lesions consistent with infantile lactic acidosis, as well as pulmonary modifications suggesting congenital alveolar dysplasia and altered hepatic architecture compatible with fibrosis.

## 1. Introduction

Polysplenia syndrome represents a type of left atrial isomerism characterized by multiple small spleens, often associated with cardiac malformations and with defects in the usual left–right distribution of the thoracic and abdominal organs. This is part of the spectrum of heterotaxy or *situs* *ambiguus* syndromes. of the abdominal organs. In most cases, the cardiac malformations are mild and not life-threatening, in contrast to right atrial isomerism characterized by asplenia and frequent severe cardiac malformations. Atrioventricular canal and septal defects, inferior vena cava abnormalities, bilateral bilobation or trilobation of the lungs, midline liver, right-sided stomach, and a degree of intestinal malrotation are the most common described findings associated with polysplenia.

The body axes, anteroposterior, dorsoventral, and right-left, are organized before and during the gastrulation phase. Early organ primordia, such as the heart and the liver, develop initially at the midline with molecular lateralization that later manifests physically as the embryo develops [1]. Blood vessels derived from the pharyngeal arches such as the aorta also start as symmetrically paired structures before regression of certain segments and remodeling under the influence of flow [2]. Other organs such lungs develop from the beginning as asymmetrical lateralized structures. Symmetry breaking begins during the process of gastrulation. Epithelial cells at the site of mesodermal ingression have cilia, which revolve rapidly in a clockwise direction. It is thought that this rotational movement acts as a patterning cue, determining local flow of nodal extra-embryonic fluid and accumulation of key molecules that affect left–right axis determination [1]. When the primitive streak appears, the fibroblast growth factor 8 (FGF8) is produced by the node at the point of mesodermal ingression to induce *NODAL* transcription factor expression on the left side of the embryo. Subsequently, during neurulation, FGF8 maintains *NODAL* expression while *LEFTY2* is also induced in the left lateral mesoderm only. These two secreted ligands of the transforming growth factor-beta (TGF-b) superfamily amplify expression of the *PITX2* transcription factor, a key homeotic determinant of left identity. However, right determinants are not as well known, although SNAIL family transcription factors may play a role in preventing random laterality specification [3,4]. Human heterotaxy syndromes are genetically heterogeneous; a current diagnostic panel screens 74 known associated genes, including *NODAL*, *LEFTY2*, and *PITX2* [5].

The case of polysplenia presented is of a one-month-old infant with severe cardiac malformations, *situs ambiguus* of the abdominal organs, and ceased development of the lung parenchyma in the form of congenital alveolar dysplasia. To our knowledge, this is the first case to describe such a range of complex anomalies.

## 2. Detailed Case Description

A full autopsy of the one-month-old infant was performed in the green-zone or non-restricted COVID-19 area of the County Clinical Emergency Hospital’s morgue. After a careful macroscopic examination of the internal organs and a detailed description of the anomalies found, photographs were taken from multiple angles and with different focal lengths for scientific purposes. Tissue samples were collected from every single organ and then fixed in 10% formalin solution. After fixation, the tissue collected was included in paraffin blocks for the intended purpose of creating microscopic hematoxylin-eosin slides. The slides were meticulously examined and described, and photographs were obtained to highlight the most significant histopathologic changes.

## 3. Results

The case is of a one-month-old male patient, weighing approximately 3000 g, born at the County Clinical Emergency Hospital of Sibiu, who was hospitalized from birth until death. He was diagnosed with agenesis of the gallbladder, situs ambiguous, congenital malformation of the pulmonary artery, multiple cardiac malformations with neonatal heart failure, and congenital malformation of the spleen. The laboratory findings included active infection with Escherichia coli, relative clotting disorder, acute renal failure, increased white blood cell count with neutrophilia, and lymphocytopenia. Prior to his death, comprehensive treatment including antibiotics, corticosteroids, and assisted oxygenation were administered. However, the patient’s condition deteriorated, he presented asystole without effective response to resuscitation, and his death was declared.

**Figure 1:**
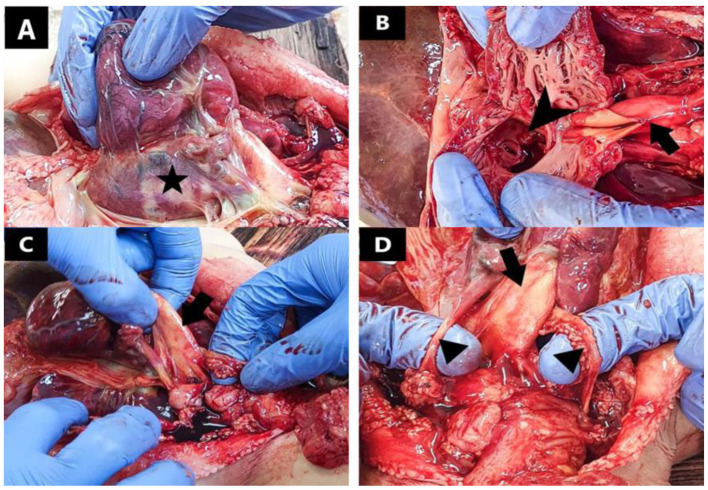
Macroscopic aspect of myocardium and great vessels: (**A**) (star) common atrium; (**B**) complete atrioventricular canal defect (arrowhead) and partially dissected common arterial trunk (arrow); (**C**) (arrow) common arterial trunk; (**D**) bilateral superior vena cava (triangle) and common arterial trunk (arrow).

Upon external examination, we observed a pale skin with red to violet lividities found on the dorsal uncompressed areas of the body and a yellow mucous secretion coming out from the nostrils. No somatic malformations were present upon external examination of the body. The front fontanelle was 6 × 3 mm. Internal examination of the cranial cavity revealed diffuse hyperemic meninges with accentuated vascular markings. The brain was of low consistency, well differentiated, forming cerebral gyri and sulci. The cerebral ventricles were slightly dilated. Upon internal examination of the thoracic cavity, we found a rounded heart, increased in volume, formed by a dilated common atrium, a hypertrophic left ventricle situated more anterior, and a hypoplastic right ventricle situated more posterior. After dissection of the heart, we concluded that the inter-atrial septum was absent, there was an interventricular septal defect directly beneath the atrioventricular junction, and the common atrium communicated with both ventricles through a joint orifice of about 15 mm in diameter (Figure 1). The anomalies identified were consistent with a complete atrioventricular canal defect.

The pulmonary venous return was supported by two right pulmonary veins, respectively two left pulmonary veins communicating with the common atrium through separate orifices. The inferior vena cava was present, with influx into the common atrium. The superior systemic venous return was sustained by two superior vena cava, one left and one right. We identified a common large caliber arterial trunk starting from the left hypertrophic ventricle which generated multiple branches, such as two pulmonary arteries, a brachiocephalic arterial trunk on the right, a common carotid, and a subclavicular artery on the left, all with superior and lateral trajectories. The common trunk had a single valve with three cusps.

**Figure 2:**
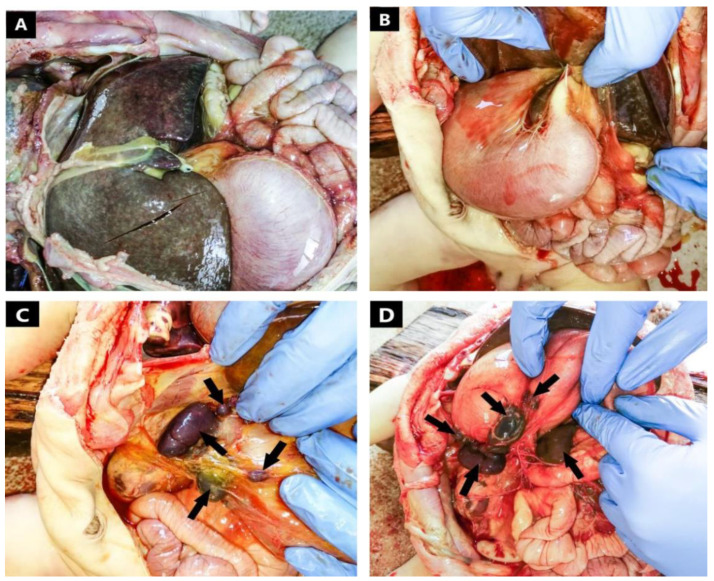
Macroscopic aspect of the abdominal cavity: (**A**) midline liver with almost symmetrical lobes; (**B**) right-sided stomach; (**C**) (arrows) multiple right sided spleens; (**D**) (arrows) multiple spleens among the greater curvature of the stomach.

Both lungs had only two lobes, suggesting bilateral left sideness of the pulmonary system. Dissecting the abdominal cavity (Figure 2), we came across a right sided dilated stomach with pale mucosa and large amount of white food content. Pancreatico-duodenal envelope was located with the convexity on the left side and the tail of the pancreas stretched to the right hypochondrium. In addition, we found complete inversion of the intestines, a midline liver, and gall bladder agenesis.

The splenic tissue was divided into one large spleen located in the right upper quadrant, immediately below the diaphragm with a long axis of two centimeters and subdivided into two distinct lobes; a small, rounded spleen with a diameter of approximately one centimeter, located inferior from the first spleen; and several smaller spleens situated among the greater curvature of the stomach and on the anterior wall. No macroscopic abnormalities were found upon proper examination of the genitourinary system.

Upon histological examination of the brain tissue sampled from the brainstem, cerebral hemispheres, and basal ganglia, there were no structural abnormalities in the neurons and glial cells. There was marked oedema of the white matter, congestion of the small vessels, and focal fresh subarachnoid hemorrhage. The epithelial lining of the ventricles had no pathological changes. The cerebellum was of normal histological stratification with preserved architecture, however there was severe meningeal and intracerebellar congestion and oedema.

**Figure 3:**
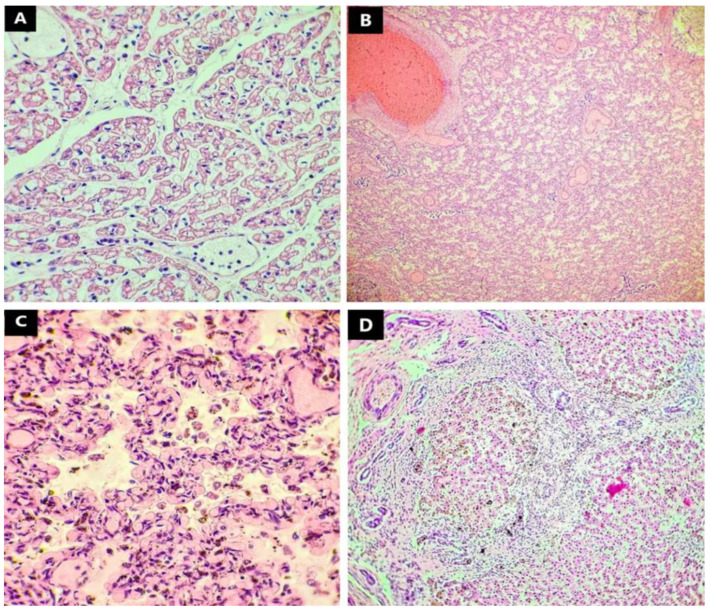
Microscopic aspect of the heart, lung, and liver—Haematoxylin Eosin (40× 100×): (**A**) (heart) (100×) large areas of myocytes expressing faintly granular expansion of the central clear zone with myofibrils showing exclusively at the periphery; (**B**) (lung) (40×), (**C**) (lung) (100×) lung parenchyma trapped in an early saccular phase with increased cellularity and prominent interstitium; (**D**) (liver) (100×) enlarged fibrotic portal tracts formed by dense collagen fibers—porto-portal bridging.

Upon microscopic examination of the prelevated lung tissues, we discovered that the infant’s lungs were severely underdeveloped. The lung parenchyme appeared to have remained in an early saccular phase with increased cellularity and prominent interstitium. There was a continuous presence of angulated distal air spaces with formation of mesenchymal crests containing capillaries (Figure 3C). The alveolar walls were lined with admixed type I and type II pneumocytes. The air spaces were filled with an eosinophilic proteinaceous acellular material and limited numbers of inflammatory cells, mainly macrophages. The lobular architecture was faint, and the blood vessels were markedly congested, with few medium size arteries showing some medial hyperplasia and intimal fibroid and myxoid thickening, consistent with a degree of pulmonary hypertension (Figure 3). However, our opinion was that the histopathological aspect appeared consistent with congenital alveolar dysplasia [6]. No capillary and venous abnormalities were discovered to suggest alveolar capillary dysplasia.

Upon detailed examination of the myocardium under the microscope, we discovered large areas of myocytes expressing faintly granular expansion of the central clear zone with myofibrils showing exclusively at the periphery, a lesion consistent with infantile lactic acidosis [7,8] (Figure 3A). The blood vessels supplying the heart had no histological abnormalities, with only small, congested vessels present, and lymphocytes within the lumen.

Microscopic examination of the liver indicated a disordered but intact lobular structure with preserved acinar architecture. There were enlarged fibrotic portal tracts formed by dense collagen fibers projected outside these areas, many of which established porto-portal bridges. There was also canalicular hyperplasia and a rich lymphocytic inflammatory infiltrate within the portal tracts. Dilatation of the portal venules was found throughout the area examined.

The spleens revealed a congested red pulp comprised of numerous erythrocytes with normal architecture and a slightly smaller white pulp composed of mature lymphocytes without cytological atypia, forming only focal lymphoid follicles, with some follicles that skirted germinal centers. Some of the central arteries had thin walls and were moderately dilated, with red blood cells in the lumen. The capsule was intact.

Upon microscopic examination of the kidney, we observed a typical number of glomeruli with normal morphology for a newborn. The proximal and distal convoluted tubules from the cortical area sustained focal tubular necrosis defined by tubules that suffered tubulorrhexis with ballooned cubic cells that had been detached from the basal membrane and focal zones of tubular epithelial whorls, areas alternating with autolysis. No histological abnormalities were found upon comprehensive examination of the stomach, small and large intestines, and adrenal glands.

After macroscopic evaluation of the body at the autopsy, we concluded that the patient suffered cardio-respiratory arrest due to severe hypoxia in association with septicemia, in the context of a series of complex cardiac malformations associated with congenital abdominal organ anomalies. The macroscopic pathological diagnosis was common atrium with complete atrioventricular canal defect, left ventricular hypertrophy, right ventricle hypoplasia, truncus arteriosus, superior vena cava duplication, bilobation of the lungs, situs ambiguous of the abdominal organs with right-sided stomach, a midline liver with almost symmetrical lobes, cholecystic agenesis, multiple right-sided spleens, and complete inversion of the intestines and pancreas (Table 1).

Histopathological evaluation of the tissues collected from every organ during the autopsy concluded that the patient suffered cardiac lesions consistent with infantile lactic acidosis. The lung parenchyma presented early saccular morphology with increased cellularity and prominent interstitium suggesting congenital alveolar dysplasia. Hepatic architecture was compatible with fibrosis presenting enlarged fibrotic portal tracts formed by dense collagen fibers and porto-portal bridging. (Figure 3; Table 1).

## 4. Discussions

After thorough evaluation of the autopsy results, we concluded that the patient suffered from polysplenia syndrome, a form of heterotaxia (situs ambiguous) with right atrial isomerism associated with multiple cardiac malformations. We found the heart to be enlarged, having a round form, primarily due to a dilated common atrium and a hypertrophic and dilated left ventricle situated more anterior, in contrast with a hypoplastic right ventricle situated more posterior. After dissecting the heart, we discovered a complete atrioventricular canal defect, which is more common in right-sided isomerism or asplenia [9,10,11]. This heart defect is also associated with polysplenia syndrome [9,12,13,14,15,16].

From the hypertrophic left ventricle arose a common arterial trunk (truncus arteriosus) from which emerged two pulmonary arteries, a brachiocephalic arterial trunk, a common left carotid artery, and a left subclavian artery. The common trunk (Table 1) continued with the descending aorta. This association between polysplenia and truncus arteriosus was also described by Hirokazu et al. [16] and furthermore it was related to cases of right isomerism or asplenia [17]. The cephalic venous drainage was sustained by a duplicated superior vena cava that drained directly into the common atrium through separate orifices. This was mainly due to a persistence of the left anterior cardinal vein and the lack of formation of the left brachiocephalic vein [2]. Most medical studies described inferior vena cava abnormalities associated with polysplenia [9,10,18,19]; however, Moller et al. found six cases describing bilateral vena cava within the polysplenia syndrome [15].

In situ examination of the lungs revealed that each lung was bilobar (Table 1), suggesting a bilateral left-sideness of the pulmonary system commonly described in the medical literature [15]. Trilobation of the lungs was also described in association with left atrial isomerism [10].

After having found the thoracic organs in situs solitus, when we reached the abdominal cavity, we observed the organs placed in situs ambiguous (Table 1). We discovered a right-sided stomach, a midline liver with almost symmetrical lobes, gall bladder agenesis, and a complete inversion of the small and large intestine as well as the pancreas. Multiple studies have described malposition of the stomach as well as a midline liver in association with polysplenia [9,10,15,20,21]. Many case studies have described malrotation of the intestines instead of complete mirror inversion [20,21,22]. The absence of the gall bladder is a rare finding in polysplenia syndrome. Moller et al. described only one case with such result [15].

The splenic tissue was divided into one large spleen subdivided into two distinct lobes, a smaller, more rounded spleen, situated inferior from the first, and several smaller spleens situated among the greater curvature of the stomach and on the anterior wall (Table 1). The topography of the multiple spleens found in our case is consistent with polysplenia syndrome [15,23,24]. The macroscopic aspect of the spleens is of normal morphology and they did not present a health issue and did not contribute to the thanatogenesis in our case.

Microscopic evaluation of collected tissues revealed no specific abnormalities consistent with polysplenia syndrome. The most significant finding in the examination of the lung parenchyma was the detection of abnormalities in the development of the alveoli. The alveolar tissue was stuck in the early saccular phase with increased circularity and prominent interstitium but with respect to the capillary and venous systems. In the early saccular phase, the gas-exchange surface area of the lungs significantly expands and the distal airways form enlarged airspaces known as saccules, separated by thick septa in which the type II pneumocytes starts maturating into type I pneumocytes [25]. The pulmonary surfactant starts producing, but, in this phase, there is not enough to maintain the inflation of the saccules, and thus our patient suffered from acute respiratory insufficiency, which is why he was on an assisted breathing device from birth until death. This finding was consistent with congenital alveolar dysplasia. While certain researchers describe a form of congenital alveolar capillary dysplasia associated with polysplenia syndrome or asplenia [26,27], to our knowledge the type of alveolar dysplasia without capillary involvement in association with polysplenia syndrome has never been reported in the medical literature.

Histopathological changes corresponding with lactic acidosis were found upon examination of the myocardium, and this alteration is most likely secondary to hypoxia, due to fluctuating oxygen levels the patient may have experienced, throughout the admission, with assisted breathing.

Only the liver presented mild fibrosis with moderate biliary hyperplasia; the periportal fibrosis was extensive, forming periportal fibrous bridges, but without presence of regenerative nodules. We attributed this histopathological change to the bile system anomalies such as gall bladder agenesis and biliary atresia.

Regarding the microscopy of the spleens, there was no pathological change; all spleens presented a congested red pulp and a slightly hypoplastic white pulp, with no presence of lymphoid follicles, and most of the lymphatic tissue was found around the central arteries. We concluded that the spleens were functional.

The pathophysiology and molecular genetics of polysplenia syndrome remain incompletely understood. Many genes encoding key proteins of the TGF-b pathway are candidates for left- or right-sided isomerism [5,28,29]. Mutation in the *CFC1* gene, encoding a component of the multi-subunit receptor for *NODAL*, is a particularly good candidate for the broad spectrum of phenotypic changes, such as polysplenia, complex cardiac anomalies, left isomerism of lungs, bilateral superior vena cava, midline liver, right-sided stomach, and intestinal malrotation, as we have described here [30]. *CFC1* mutation have also been found in 80% of cases with left isomerism according to Loomba et al., while *NODAL* mutation was more consistent with right isomerism [31]. Overall, the exact genetic mechanism that triggers polysplenia syndrome is still largely unknown, requiring more frequent diagnosis of ante- or post-mortem cases.

## 5. Conclusions

Polysplenia syndrome remains a vast and sometimes lethal congenital abnormality, which may remain under diagnosed, even after an autopsy is performed. This may be due to the limited number of cases identified and documented so far; therefore, this diagnosis may be underestimated by medical practitioners and pathologists. To add to the complexity of the polysplenic syndrome, cardiac malformations are likely present. These are sometimes mild, but in other situations they can be extremely severe with almost no chance of survival.

Such presentations require thorough examination and analysis, in particular examination of all specific abnormalities associated with it, followed by consistent reporting of the cases. We think it is important to encourage many other case reports to be made, in order to apprehend the entire spectrum of morphological anomalies across all organs. To our knowledge, this is the first case describing such wide range of malformations, but it is unlikely to be the last.

## Figures and Tables

**Table 1:** Macroscopic and microscopic findings after autopsy and histopathologic examination.

Organ/System	Macroscopic Findings	Microscopic Findings
Cardiovascular system	Common atrium	Infantile lactic acidosis
	Complete atrio-ventricular canal defect	
	Left ventricle hypertrophy	
	Right ventricle hypoplasia	
	Common arterial trunk	
	Superior vena cava duplication	
Pulmonary system	Bilateral left-sideness of the pulmonary system (bilobation of the lungs)	Congenital alveolar dysplasia
Hepatobiliary system and pancreas	Midline liver	Hepatic fibrosis
	Symmetrical hepatic lobes	
	Gall bladder agenesis	
	Inversion of the pancreas	
Digestive tract and spleen	Right-sided stomach	-
	Complete inversion of the intestines	
	Multiple right-sided spleens	
Uro-genital system	-	-

## Data Availability

Not applicable.
